# Identification of LncRNA Prognostic Markers for Ovarian Cancer by Integration of Co-expression and CeRNA Network

**DOI:** 10.3389/fgene.2020.566497

**Published:** 2021-02-16

**Authors:** Huisheng Liang, Yuquan Bai, Hailong Wang, Xiangjun Yang

**Affiliations:** ^1^Department of Gynecology and Obstetrics, The Affiliated Zhongshan Hospital of Xiamen University, Xiamen, China; ^2^Organ Transplantation Institute, School of Medicine, Xiamen University, Xiamen, China; ^3^Department of Thoracic Surgery, West China Hospital of Sichuan University, Chengdu, China; ^4^Department of Basic Medicine, School of Medicine, Xiamen University, Xiamen, China

**Keywords:** immune cell abundance, weighted gene co-expression network analyses, long noncoding RNA, ceRNA network analysis, ovarian cancer

## Abstract

**Background:**

Ovarian cancer (OC), one of the most prevalent gynecological malignancies, is characterized by late detection and dismal prognosis. Recent studies show that long non-coding RNAs (lncRNAs) in competitive endogenous RNA (ceRNA) networks influence immune infiltration and cancer prognosis. However, the function of lncRNA in OC immune infiltration and prognosis remains unclear.

**Methods:**

Transcriptomes of 378 OC samples and clinical data were retrieved from the TCGA repository. Modules related to immune cells were identified using weighted gene co-expression network analysis (WGCNA). Functional enrichment analysis and survival analysis were then performed for the identification of immune-related lncRNAs in the brown module using Cox regression model. Finally, a ceRNA network was constructed by using the lncRNAs and mRNAs from the brown module.

**Results:**

We found lncRNAs and mRNAs in the brown module to be significantly associated with immune cells in OC and identified 4 lncRNAs as potential OC prognostic markers. We further established that lncRNAs in the ceRNA network influence OC immune infiltration and prognosis by regulating miRNA, ultimately modulating mRNA levels.

**Conclusion:**

We have identified 4 lncRNAs as independent immune prognostic factors for OC. Furthermore, our findings offer novel insight into lncRNAs as OC immune and prognostic biomarkers.

## Introduction

Ovarian cancer (OC) is one of the most prevalent gynecological cancers, with more than 200,000 new cancer cases and 125,000 fatalities annually (Zhao et al., 2016). OC is often diagnosed at advanced stage and is associated with dismal prognosis. Approximately 75% of recurrent OC cases are incurable (Lheureux et al., 2019), and its 5 years survival rate is < 45% (Webb and Jordan, 2017). Despite tremendous advances in cancer treatment, OC survival remains poor (Shuang et al., 2015). Given that accurate and effective biomarkers may improve early cancer diagnosis and overall survival (Jin et al., 2015), we sought to identify potential biomarkers.

Immunotherapy is an effective anticancer treatment (Rodriguez et al., 2018; Shen et al., 2019; Farkkila et al., 2020). Immune cells have also been reported to affect OC prognosis (Drakes and Stiff, 2018; Cheng et al., 2020; Jiang et al., 2020). Therefore, understanding immune cell infiltration in ovarian tissue may help determine the specific role of immune modulation in OC (Kulbe et al., 2012; Reinartz et al., 2014; Montfort et al., 2020). Currently, the role of lncRNA in immune modulation is not clear and deserves further exploration (Fok et al., 2018; Yu et al., 2018; Yang C. et al., 2019).

Long non-coding RNAs (lncRNAs) are comprised of > 200 nucleotides (Zhang et al., 2019), and modulate cancer cell proliferation in tumors either through nucleotide mutation or gene expression alteration (Lv et al., 2020). Although a large number of lncRNAs are known, their role in the immune modulation of OC is unclear (McAninch et al., 2017). There have been many studies on the prognosis-related lncRNA of OC; for example, Li and Zhan (2019) identified 16 survival-related lncRNAs and TF-lncRNA STAT3-FOS has been proved to affect the prognosis of OC by Guo et al. (2020). More useful and potential lncRNA should be explored as a prognostic marker for OC, so that there are more possibilities to find the most effective cancer treatment targets.

Competitive endogenous RNA (ceRNA) is a model regulatory network in which RNA species mutually modulate each other via competitively shared miRNA response elements (Li and Zhan, 2019; Liu H. et al., 2019). In ceRNA networks, lncRNAs interact with mRNA by competitively binding miRNA. Besides advancing our understanding of mRNAs and lncRNAs regulatory networks, the discovery of ceRNA has offered new perspectives of exploring the incidences of various cancers (Profumo et al., 2019; Ye et al., 2019; Yue et al., 2019).

Weighted gene co-expression network analysis (WGCNA) clusters genes by gene expression (Yang M. et al., 2019) and has previously been used to study biological systems networks. This approach elucidates regulatory correlation between genes and identifies new modules (Pol et al., 2017; Favreau et al., 2019).

Here, we retrieved lncRNA and mRNA expression profiles from TCGA and used WGCNA to identify lncRNAs associated with OC immune modulation and prognosis.

## Materials and Methods

### Data Collection and Processing

TCGA OC transcriptome datasets on OC were retrieved from the GDC data portal^1^. Level 3 RNA-seq V2 (including lncRNA and mRNA expression data) and clinical data for 378 OC patients were analyzed. The sequencing data of normal ovarian tissue was obtained from the Genotype-Tissue Expression (GTEx) database^2^. In addition, the data for the validation set (GSE26193 and GSE63885) came from the NCBI Gene Expression Omnibus (GEO)^3^. Prior to bioinformatics analysis, we used microarray annotation data to match probes with analogous gene IDs. Genes with multiple deleted expression values (expression level = 0, and 20% more) were excluded. These data were obtained from open access resources, thus ethical approval was not required.

### Evaluation of Tumor-Infiltrating Immune Cells (TIICs)

We used CIBERSORTx^4^ (Newman et al., 2019), an online analytical tool that utilizes RNA expression data to determine the proportion of specific cells, to determine the proportion of 22 TIICs in the dataset. We used R packages to visualize the results and evaluate correlation between immune cells and prognosis.

### Weighted Correlation Network Analysis of lncRNAs and mRNAs

WGCNA was used to examine correlation between co-expression modules and immune cells infiltration. We processed the OC TCGA data and selected the top-2,500 lncRNAs and top-2,500 mRNAs based on RNA expression variance. The relationship between corresponding samples and immune cells infiltration was visualized using the WGCNA R package (Langfelder and Horvath, 2008). After selecting an optimal soft threshold, a weighted gene co-expression network was constructed using a suitable scale-free character of biological gene networks. Next, a co-expression network was summarized in a cluster dendrogram based on gene correlation. Association between the co-expression module and infiltration by three immune cells was presented as a Pearson correlation coefficient and visualized on a heatmap using the R Heatmap tool package. Correlation between sample traits was evaluated by calculating genetic significance (GS) and module significance (MS) based on the WGCNA results.

### CeRNA Network Construction and Analysis

WGCNA results were used to construct the ceRNA network based on total lncRNAs and mRNAs in the most relevant modules. First, the miRcode repository^5^ (Jeggari et al., 2012) was used to predict interaction between lncRNAs and miRNA. Next, we predicted target mRNAs for miRNAs using TargetScan^6^ (Lewis et al., 2005), miRDB^7^ (Wong and Wang, 2015), and miRTarBase^8^ (Hsu et al., 2014). Intersections between target mRNAs and mRNAs in the brown module were then selected for subsequent analysis. Finally, Cytoscape 3.7.2^9^ (Shannon et al., 2003) was used to create and visualize the lncRNA–miRNA–mRNA ceRNA network based on the above findings.

### Gene Ontology, Pathway, and Function Enrichment Analysis

The R packages clusterProfiler (Yu et al., 2012) and GOplot were used for GO term and KEGG enrichment analyses. Additionally, function enrichment analysis was performed within the mRNA network to identify OC-related functions using GenCLiP3^10^ (Wang J. H. et al., 2019), which explores functions and regulatory networks of human genes on PubMed.

### Construction of Prognostic Signature Within the Brown Module

Prognostic data were generated on the RNAs matrix involved in the brown module and matched with follow-up data. First, we screened for lncRNAs and mRNAs related to OC prognosis using univariate Cox regression analysis (Stel et al., 2011). Least absolute shrinkage and selection operator (LASSO) Cox regression analysis was then used to determine the prognostic value of the lncRNAs and mRNAs. Based on the LASSO Cox regression results and the expression levels of the lncRNAs and mRNAs, we generated a risk score for each patient. Patients were then divided into a high- and low-risk group based on their respective scores relative to the median risk score. A log-rank test was used to compare survival between the two groups using Kaplan-Meier analysis. Finally, receiver operating characteristic (ROC) analysis was used to estimate the signature’s predictive power for 3 and 5 years periods.

### Identification of Immune-Related lncRNAs in OC

We used ImmLnc^11^ (Li et al., 2020), an online tool used to elucidate relationships between lncRNAs and immune cells, to identify immune-related lncRNAs in the OC dataset. Statistical significance was set at *p* ≤ 0.05.

### RNA Extraction and qRT-PCR

Total RNA was, respectively, obtained from ovarian cells (IOSE80, Anglne, OVCAR-3, A2780) by standard TriZol method. In simple terms, 1 ml TriZol was added into samples for dissolution through 5 min incubation. Then, 200 μl chloroform was involved into samples and mixed vigorously, followed by separation with 12,000 r/min centrifugation. The RNA components in supernatant were extracted with isopropanol and then washed with 75% ethanol. RNA pallets were dissolved with RNase-free H_2_O. RT-PCR (revers-transcription PCR) was performed to generate cDNA from RNA. qRT-PCR reaction system contained 5 μl SYBR buffer, 4 μM primers (forward and reverse primers), 2 μl RNasefree water, and 1 μl cDNA. Beta-actin was set as an internal control for gene quantification. The numbers of technical and biological replicates were at least three times for each gene with qRT-PCR analysis. The primer sequences for qPCR were LINC00525, 5′-GAAACAAGATTCACAAGTGAGG-3′, 5′-AAGTCTTCTGTCTCTGATTCAG-3′. AL360004.1, 5′-GGC TCAGCTACTGAAGCCGG-3′, 5′-AGGGGCCTGGCTGTCCT GCT-3′. TLR8-AS1, 5′-TTTGCTCACTGCAACATCC-3′, 5′-CG CCTACATCTGTAGTCCC-3′. LINC00402, 5′-AAGTGGATATG GAAGCTTGG-3′, 5′-CGGAATAACAATCTGAAGATGG-3′.

### Statistical Analysis

The original data were downloaded from TCGA and GEO datasets and analyzed by R software 4.2 with WGCNA package. For the pair of module–trait relationship and gene significance (GS) for module membership (MM) based on WGCNA analysis, Pearson correlation coefficient (r) was calculated. Benjamini–Hochberg for multiple testing and false discovery rate (FDR) were used to correct the *p*-value, while *p*-value for GO enrichment analysis of mRNAs in mRNA-based brown co-expression module was obtained by two-sided hypergeometric test and corrected by Benjamini–Hochberg. Each experiment for qRT-PCR was repeated in totality three times, and the means and standard deviations (mean ± SD) were calculated. The differences between groups for *in vitro* studies were analyzed by *t*-test in SPSS 25.0 (SPSS Inc., Chicago, United States), with statistical significance (*p* < 0.05).

## Results

### Evaluation of Tumor-Infiltrating Immune Cells (TIICs)

**FIGURE 1:**
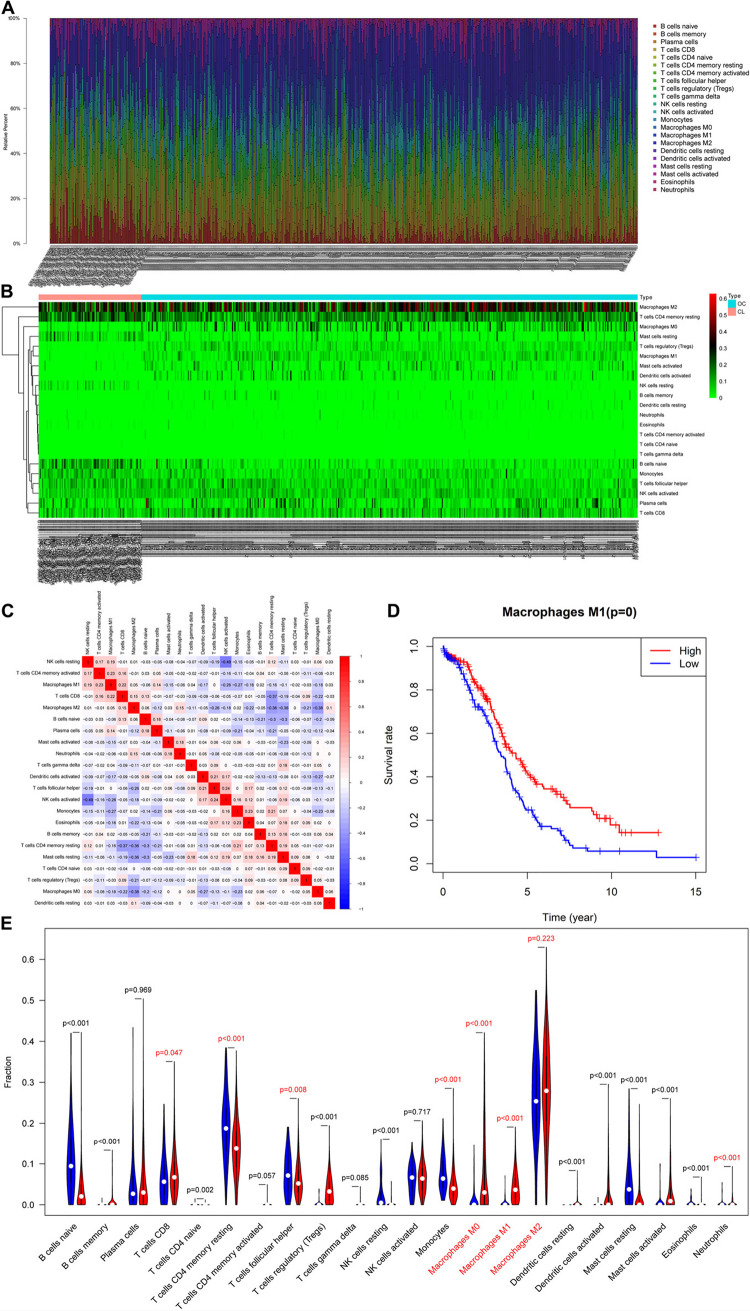
Evaluation of tumor-infiltrating immune cells (TIICs). **(A)** Relative percentage of 22 subpopulations of immune cells in 378 OC samples. **(B)** Heatmap of 22 subpopulations of immune cells in 378 OC samples. **(C)** Heatmap of correlations between infiltrated immune cells in OC. **(D)** Survival plots of the proportion of M1 macrophages in OC samples. **(E)** The difference in the proportion of immune cells between ovarian cancer and normal ovarian tissue.

After pre-processing the TCGA and GTEx data, we obtained 466 RNA expression matrix samples (including 378 OC samples and 88 normal ovarian tissue samples). Analysis of the proportion of immune cells in each sample using CIBERSORTx revealed marked differences in the proportion of immune cells in each sample (Figures 1A,B). The proportion of 22 TIICs in tumors had a weak–strong correlation. The positive correlation between M1 macrophages and activated memory CD4 T-cells was strongest (Pearson correlation = 0.23), while resting NK cells and activated NK cells had the strongest negative correlation (Pearson correlation = 0.49) (Figure 1C). Analysis of the relationship between immune cells and prognosis revealed that the proportion of M1 macrophages in OC samples was significantly associated with OC prognosis (Figure 1D). As shown in Figure 1E, whether in OC tissue or normal ovarian tissue, M2 macrophages accounted for the main proportion. And the proportion of M0 and M1 macrophages in OC tissues were significantly higher than the figure in the normal (*p* < 0.001). Thus, we speculated that macrophages were significantly associated with OC prognosis and focused our attention on M0, M1, and M2 macrophages.

### Construction of Co-expression Modules of OC

**FIGURE 2:**
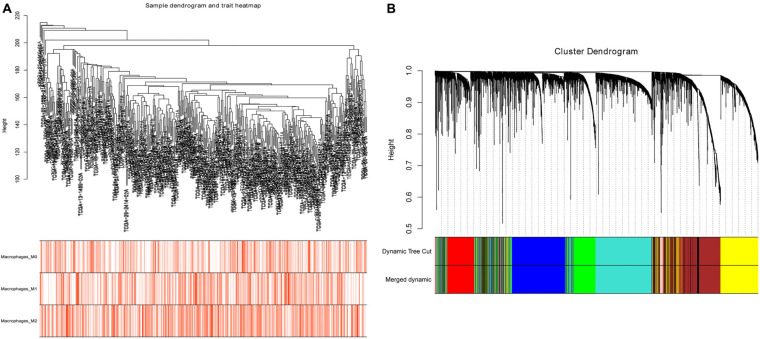
Sample cluster analysis and construction of co-expression modules based on OC RNA dataset. **(A)** Sample dendrogram and trait heatmap based on lncRNA and mRNA expression data and immune cell infiltration data. **(B)** Clustering dendrograms of lncRNAs and mRNAs, with dissimilarity based on topological overlap, together with assigned module colors.

After evaluating the tumor-infiltrating immune cells, the proportion of immune cells with high prognostic correlation in each sample was used as trait data of WGCNA. Datasets containing 2,500 lncRNAs and 2,500 mRNAs were selected for WGCNA analysis, and FlashClust used to cluster the samples. Based on RNA expression and immune cell infiltration data, we constructed a sample dendrogram and a trait heatmap where each sample was divided into separate clusters to visualize classification of the immune cell infiltration data (Figure 2A). We found soft threshold power as the most influential parameter interfering with the average connectivity and independence of each co-expression module. To obtain a relative balance between scale independence and average connectivity, we analyzed network topology using soft threshold powers of 1–20. After multiple fitting tests, an optimal β-value was obtained, with the minimum number of genes in each module being 30. The MEDissThres parameter was set at 0.35 to merge closely related modules into larger modules. Adjacency matrix was used to plot the cluster-dendrogram of the selected genes, where different co-expression modules were presented in different colors. Here, lncRNAs and mRNAs were divided into nine different modules (Figure 2B) with the brown module containing 864 RNAs.

### Gene Co-expression Modules Correspond to Clinical Traits

**FIGURE 3:**
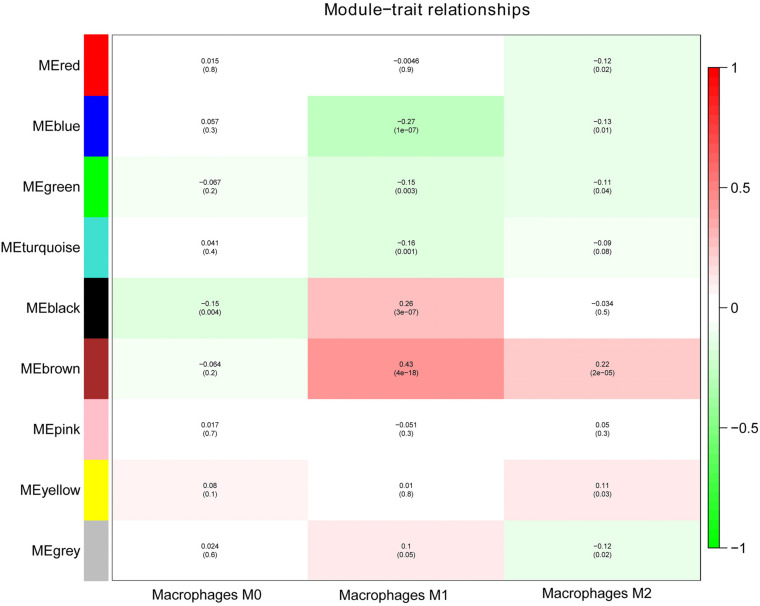
Analysis of module-trait relationships of OC based on lncRNA and mRNA data.

**FIGURE 4:**
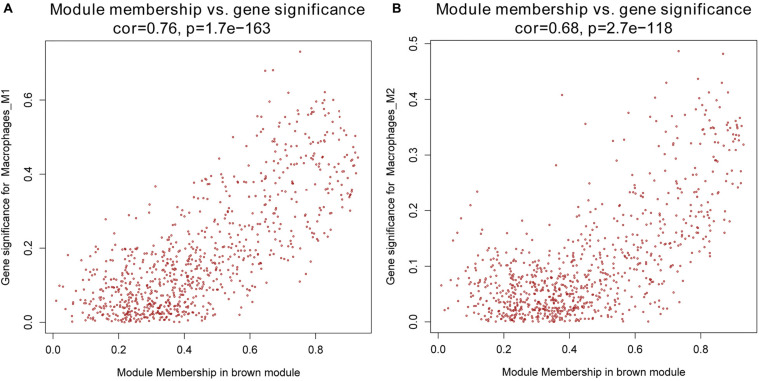
**(A)** Scatterplot of gene significance (GS) for M1 macrophages vs. module membership (MM) in the brown co-expression module. **(B)** Scatterplot of GS for M2 macrophages vs. MM in the brown co-expression module.

Next, we performed a correlation analysis of co-expression modules with specific immune cell infiltration traits in the samples. Immune cell infiltration traits included M0, M1, and M2 macrophage abundance. Heatmaps of correlation between OC immune cell infiltration features and modular genes were then depicted. Based on the module-trait association heatmap, the brown module (Figure 3) was markedly associated with M1 (*R* = 0.42, *p* = 4e–18) and M2 macrophages (*R* = 0.22, *p* = 2e–5). Besides, the scatterplots between gene significance (GS) and module membership (MM) were constructed according to the brown module. Among the modules associated with the traits of interest, genes with a high degree of module membership (MM) had greater gene significance (GS), implying that the genes in the co-expression brown modules are strongly associated with immune cells’ abundance. MM in the brown module significantly correlated with M1 (cor = 0.76, *p* = 1.7e–163) and M2 macrophages (cor = 0.68, *p* = 2.7e–118) (Figure 4).

### Gene Ontology and Pathway Enrichment Analysis in the Brown Module

**FIGURE 5:**
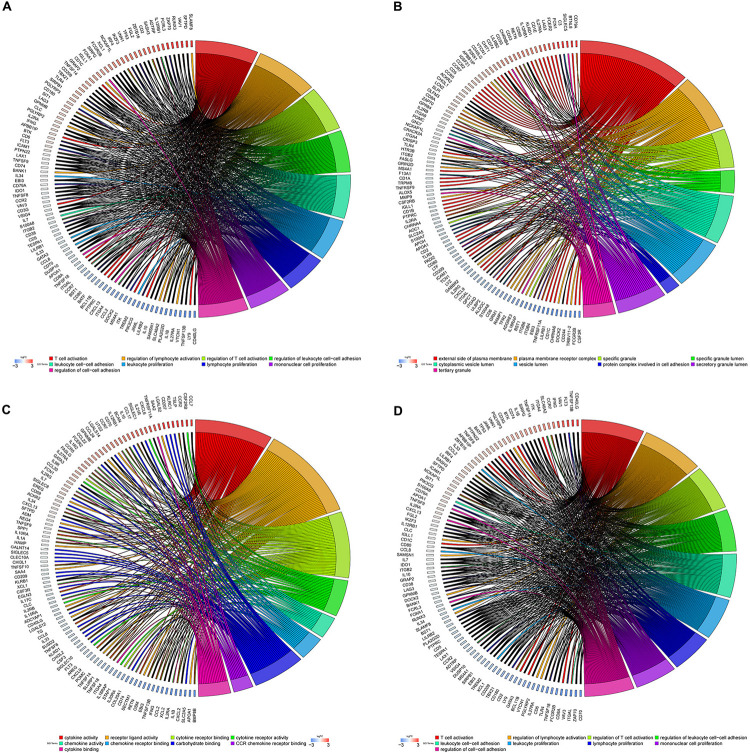
GO and pathway enrichment analysis in the brown module. **(A)** Biological process. **(B)** Cellular component. **(C)** Molecular function. **(D)** KEGG pathways. Chord plot displays relationship between genes and terms.

To further illustrate the biological function of mRNA in OC, we performed functional enrichment analysis of mRNAs in the brown module. GO term enrichment analysis revealed significant association between the mRNAs in the brown module and T-cell activation, regulation of lymphocyte activation, regulation of leukocyte cell–cell adhesion, leukocyte proliferation, protein complexes involved in cell adhesion, cytokine activity, and chemokine activity (Figures 5A–C). Additionally, pathway enrichment analysis suggested that these genes are involved in multiple immune signaling pathways, including JAK-STAT, NF-kappa B, Chemokine, IL-17, T cell receptor, and B cell receptor signaling pathways (Figure 5D), suggesting that genes in the brown module significantly correlate with immune responses in OC.

### Construction of Prognostic Signature Within the Brown Module

**FIGURE 6:**
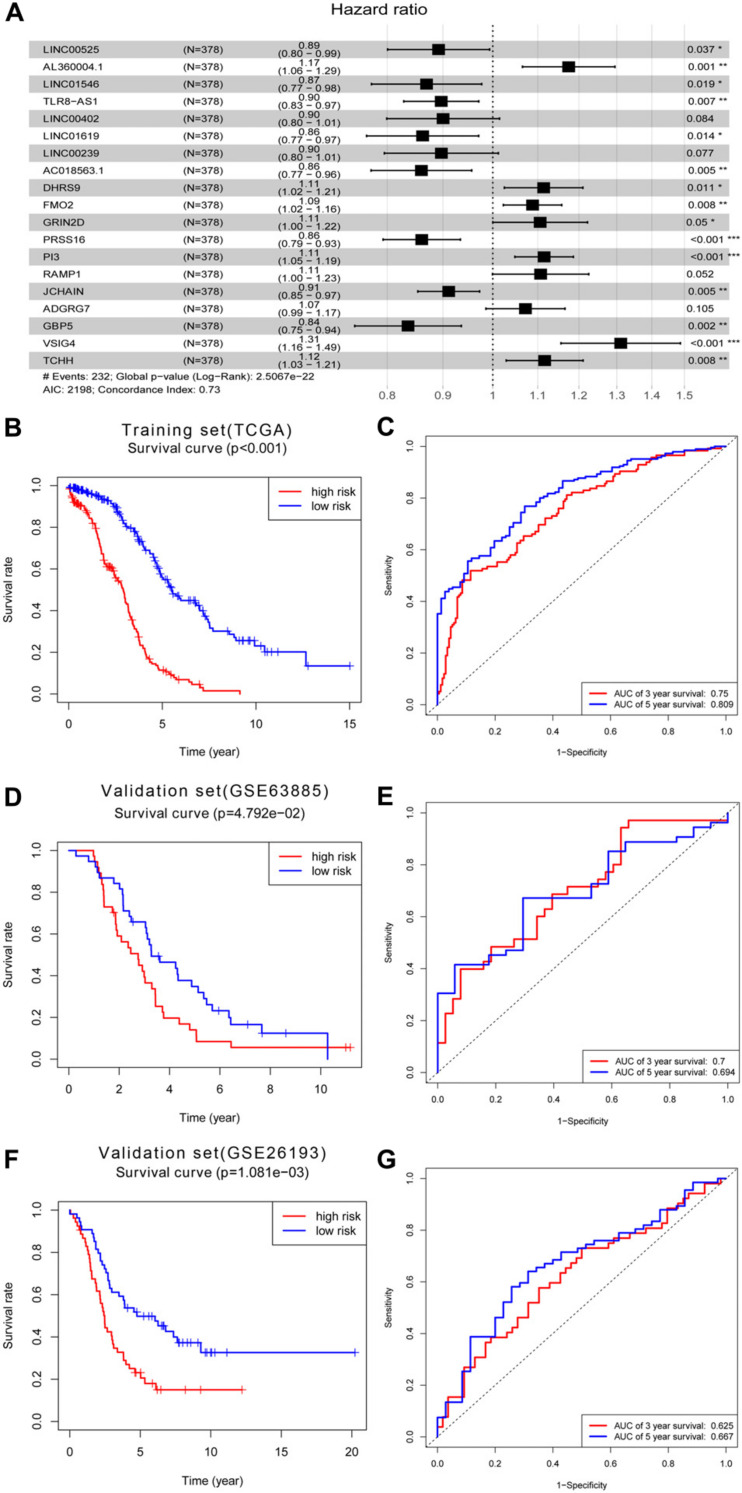
Construction of prognostic signature within the brown module. **(A)** Forest map of the optimal prognostic feature in multivariate analysis. **(B)** Kaplan–Meier survival analysis of the risk score for overall survival and **(C)** ROC analysis for the prediction of 3 and 5 years survival based on risk score in training set of TCGA. **(D)** Kaplan–Meier survival analysis of the risk score for overall survival and **(E)** ROC analysis for the prediction of 3 and 5 years survival based on risk score in validation set of GSE63885. **(F)** Kaplan–Meier survival analysis of the risk score for overall survival and **(G)** ROC analysis for the prediction of 3 and 5 years survival based on risk score in validation set of GSE26193.

Next, we used univariate Cox regression analysis to screen for prognostic genes in the brown module and uncovered 95 genes that correlated with OC prognosis (Supplementary Table 1). LASSO Cox regression analysis of these 95 genes (38 lncRNAs and 57 mRNAs) identified an optimal prognostic feature comprised of 8 lncRNAs and 11 mRNAs (Figure 6A). Risk scores were calculated to determine the prognostic risk of these 9 lncRNAs and 11 mRNAs using the formula: Risk score = expLINC00525 × 0.11460969 + expAL360004.1 × 0.160649906 + expLINC01546 × 0.140022493 + expTLR8-AS1 × 0.108942731 + expLINC00402 × 0.105005182 + expLINC01619 × 0.148341118 + expLINC00239 × 0.108698578 + expAC018563.1 × 0.150951433 + expDHRS9 × 0.107623718 + expFMO2 × 0.084195962 + expGRIN2D × 0.100325507 + expPRSS16 × 0.150213535 + expPI3 × 0.108704599 + expRAMP1 × 0.10122692 + expJCHAIN × 0.09315171 + expADGRG7 × 0.069155126 + expGBP5 × 0.179217192 + expVSIG4 × 0.270659479 + expTCHH × 0.109827863 (note: expLINC00525 = expression level of LINC00525, and so on). Based on the critical value of the median risk score, patients were divided into low- and high-risk groups. Kaplan–Meier analysis showed that the survival rate of OC patients in the high-risk group was worse relative to the low-risk group (Figure 6B). To determine the predictive effect of the 19-gene signature, time-dependent ROC curve analysis and the area under the curve (AUC) value were used as indicators. This analysis revealed a 3 years AUC of 0.75 and a 5 years AUC of 0.809, highlighting the great potential of this feature in predicting OC prognosis (Figure 6C). Based on the above 19-gene signature, we validated the model on two data sets, GSE63885 and GSE26193. Similarly, it can be found that the survival rate of OC patients in the high-risk group was worse relative to the low-risk group in the GSE63885 dataset (*p* = 0.04792, Figure 6D) and the GSE26193 dataset (*p* = 0.001081, Figure 6F). Besides, a 3 years AUC of 0.7 and a 5 years AUC of 0.694 were revealed in GSE63885 (Figure 6E), and in the GSE26193 dataset, the 3 years AUC was 0.625 and the 5 years AUC was 0.667 (Figure 6G).

### CeRNA Network in OC

**FIGURE 7:**
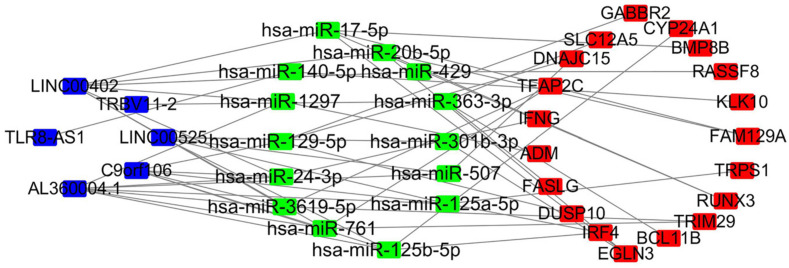
LncRNA–miRNA–mRNA network based on the brown module. Blue, lncRNA; green, miRNA; red, mRNA.

First, we used miRcode online analysis to match potential miRNAs to prognosis-related lncRNAs in the brown module. Next, TargetScan, miRDB, and miRTarBase were used to predict miRNA target genes. Comparison of the predicted target genes to mRNAs in the brown module identified 6 lncRNAs (LINC00525, C9orf106, AL360004.1, TLR8-AS1, LINC00402, TRBV11-2), 14 miRNAs (FAM129A, TRPS1, RUNX3, TRIM29, BCL11B, EGLN3, IRF4, DUSP10, FASLG, ADM, IFNG, TFAP2C, DNAJC15, SLC12A5, GABBR2, CYP24A1, BMP8B, RASSF8, KLK10), and 19 mRNAs miRNAs (hsa-miR-20b-5p, hsa-miR-125a-5p, hsa-miR-17-5p, hsa-miR-3619-5p, hsa-miR-363-3p, hsa-miR-761, hsa-miR-125b-5p, hsa-miR-129-5p, hsa-miR-1297, hsa-miR-24-3p, hsa-miR-301b-3p, hsa-miR-507, hsa-miR-429, hsa-miR-140-5p). Next, a ceRNA network of lncRNAs regulating mRNAs by binding to miRNAs was constructed (Figure 7).

### Functional Enrichment Analysis in the ceRNA Network

**FIGURE 8:**
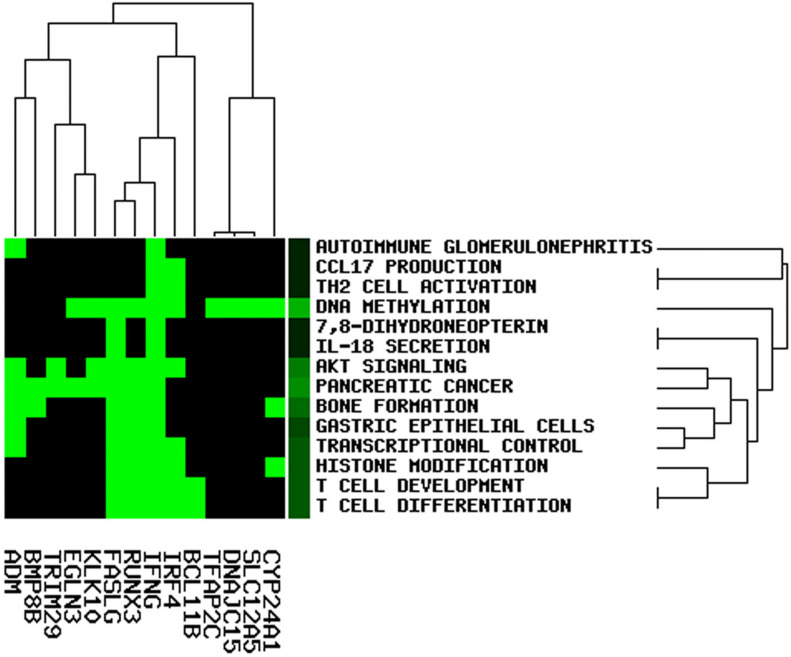
Function enrichment analysis involved in the ceRNA network.

To elucidate the potential role of lncRNA in the OC ceRNA network, we performed functional enrichment analysis using the GenCLiP3 online tool. Based on the analysis of 19 mRNAs in the ceRNA network, 14 statistically significant keywords were identified (Figure 8). These results established five different clusters, suggesting that OC might be associated with multiple biological functions. Notably, the keywords included T-cell differentiation, T-cell development, DNA methylation, histone modification, TH2 cell activation, CCL17 production, IL-18 secretion, and pancreatic cancer, all of which participate in either immune regulation or cancer.

### Identification of Immune-Related lncRNAs in OC

We next used the ImmLnc online tool to assess if lncRNAs have essential roles in OC cancer-immune cells. Evaluation of the relationship between lncRNAs and immune cells found that lncRNAs, including LINC00525, C9orf106, TLR8-AS1, LINC00402, and TRBV11-2, are associated with B-cells, CD4^+^ T-cells, CD8^+^ T-cells, dendritic cells, macrophages, and neutrophils in OC (Table 1).

**TABLE 1:** LncRNAs in the ceRNA network associated with immune cells in OC.

lncRNA	Immune cell	*p*-value	RS-value
LINC00525	CD8_Tcell	<0.001	0.188
	CD4_Tcell	<0.001	0.203
	Dendritic	<0.001	0.212
	Neutrophil	<0.001	0.257
	Macrophage	0.029	0.113
C9orf106	CD4_Tcell	<0.001	0.19
	Dendritic	<0.001	0.223
	Neutrophil	0.001	0.166
	CD8_Tcell	0.001	0.167
	Macrophage	0.013	0.128
TLR8-AS1	Neutrophil	<0.001	0.185
	Dendritic	0.001	0.179
	CD4_Tcell	0.005	0.146
	B_cell	0.012	0.13
LINC00402	Neutrophil	0.014	0.127
	CD8_Tcell	0.02	0.12
	CD4_Tcell	0.021	0.12
	Dendritic	0.033	0.111
TRBV11-2	Macrophage	<0.001	0.194
	Neutrophil	<0.001	0.333
	CD4_Tcell	<0.001	0.339
	CD8_Tcell	<0.001	0.382

**RS, rank score.**

### qRT-PCR Confirmed the Identified lncRNAs

**FIGURE 9:**
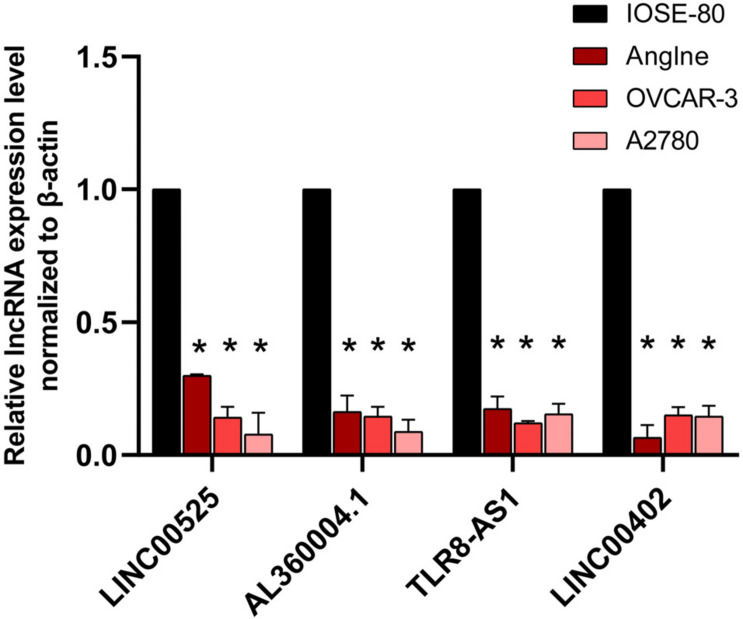
qRT-PCR analysis of four survival-related lncRNAs in OC cell models compared with control cells. *p < 0.05. *n* = 3.

qRT-PCR was used to validate the expressions of OC survival-associated lncRNAs including LINC00525, AL360004.1, TLR8-AS1, and LINC00402. Among them, all four lncRNAs were quantified with qRT-PCR. The results showed that significant difference was found for the four survival associated lncRNAs between OC cells (Anglne, OVCAR-3, and A2780) and control cell IOSE80 (*p* < 0.05) (Figure 9).

## Discussion

There is an urgent need for effective prognostic markers to improve OC overall survival (Powell et al., 2005; Shen et al., 2017; Tong et al., 2019; Zhao et al., 2019). Immune cells can enhance anticancer immune responses by recognizing cancer antigens, indicating that they may influence cancer prognosis (Drakes and Stiff, 2018). Immune infiltration in 378 OC samples and 88 normal ovarian tissue samples was analyzed and found a significant correlation between M0, M1, and M2 macrophages and OC prognosis. The proportion of M0 and M1 macrophages in OC tissues were much higher than that in the normal tissues (*p* <0.001). This was in line with previous findings (Yousefzadeh et al., 2020). OC cases with a higher proportion of M1 macrophages had a better overall survival (Macciò et al., 2020). However, M2 macrophages exhibited adverse effects in OC (Badmann et al., 2020; Nowak and Klink, 2020). These results provide deeper insights into immune infiltration in OC.

Besides influencing cancer immune modulation, studies show that lncRNAs also influence OS (Qi and Du, 2013; Wu et al., 2016; Bo et al., 2018). Additionally, lncRNAs have potential as therapeutic targets and prognostic markers in OC (Li and Zhan, 2019). The ceRNA network also contributes significantly to tumor development and progression. In the network, lncRNAs regulate target mRNA (Rutnam et al., 2014; Tang et al., 2014; Wang P. et al., 2017). WGCNA analysis of lncRNA and mRNA datasets found that the brown module significantly correlated with immune cells in OC, especially M1 and M2 macrophages. Functional enrichment analysis of the mRNA from the brown module further revealed that T-cell activation, leukocyte migration, IL-17 signaling, JAK-STAT signaling, T-cell receptor signaling, and B cell receptor signaling pathways are critical in OC. Presence of IL-17 in the tumor microenvironment can improve OC prognosis (Lan et al., 2013; Bilska et al., 2020). Interestingly, TNF-α regulates the immune system via IL-17 to promote tumor proliferation (Charles et al., 2009). JQ1 and cisplatin are targeted in the JAK-STAT signaling pathway to improve drug resistance of OC (Bagratuni et al., 2020). Further, functional analyses found that multiple immune signaling pathways are concentrated on the OC module. Thus, the immune-related lncRNAs in the brown module may be therapeutically targeted against tumors.

Univariate Cox regression survival analysis of the brown module revealed 95 prognosis-related genes (38 lncRNAs and 57 mRNAs). An optimal prognostic feature comprised of 8 lncRNAs and 11 mRNAs was obtained after multivariate Cox regression analysis. The 5 years AUC was 0.809 and the 3 years AUC was 0.75, highlighting the feature’s potential to predict OC survival. Based on this optimal prognostic feature, a 3 years AUC of 0.7 and a 5 years AUC of 0.694 were revealed in GSE63885, and in the GSE26193 dataset, the 3 years AUC was 0.625 and the 5 years AUC was 0.667. In the training set (TCGA) and validation set (GSE63885 and GSE26193), the survival time of high-risk and low-risk features are significantly different. In general, in the validation set GSE63885 and GSE26193, we received similar prediction effects as the training set, which showed that our prognostic feature had good predictive power. Previously, risk regression models of lncRNA related to the prognosis of OC were established based on the TCGA datasets, for example, the AUC at 5 years was 0.75 for the 10-lncRNAs signature established by Xu et al. (2019), 0.694 for the 8-lncRNAs signature established by Zhou et al. (2016b), and 0.705 for the 10-lncRNAs signature established by Zhou et al. (2016a). In comparison, the 5 years AUC of 0.809 established by us has shown a more excellent effect. We also used the prognosis-related genes (lncRNA and mRNA) revealed by the univariate analysis to create a ceRNA network. In the ceRNA network, 6 lncRNAs affect immune cells and OC prognosis by modulating 19 mRNAs. Of the 6 lncRNAs in the ceRNA network, multivariate Cox regression analysis identified four (LINC00525, AL360004.1, TLR8-AS1, LINC00402) as independent prognostic factors. LINC00525 regulates immune response via plasma cells in periodontitis (Wu et al., 2020), while in non-small cell lung cancer, LINC00525 regulates cancer proliferation via the ceRNA network (Yang Z. et al., 2020). Up-regulation of TLR8 and activation of NF-κB signaling has been shown to promote OC metastasis and chemoresistance via TLR8-AS1 (Xu et al., 2020). LINC00402 might also affect metastatic melanoma prognosis via the ceRNA network (Wang L. X. et al., 2019). A significant difference has also been reported between colorectal cancer patients and healthy controls with regard to the immunological indicator TRBV11-2 (Liu X. et al., 2019). Furthermore, TRBV11-2 is associated with CD8^+^ T-cell immune responses in dengue virus infection (Culshaw et al., 2017). These 4 lncRNAs as prognostic markers for OC seem to be rarely mentioned. Li and Zhan (2019) identified clinical trait—related lncRNA and mRNA biomarkers with weighted gene co-expression network analysis as a useful tool for personalized medicine in OC, while Xu et al. (2019) established a model with 10-lncRNA signature that was also compatible with patients with or without BRCA1/2 mutations and had the potential to predict the response to platinum-based adjuvant chemotherapy. Our study differed from the previous ones, and we identified the prognostic-related lncRNA based on the immune cell infiltration related to the prognosis of OC. In this way, the lncRNA we obtained many affect the prognosis of OC by regulating immune cells M1 macrophages. This would be the basis for us to apply these lncRNAs as targets for OC immunotherapy. We speculate that the lncRNAs in the ceRNA network may serve as immune prognostic markers for OC. The results provide new insights into immune diagnostic markers and prognostic detection factors for OC.

LncRNAs adversely affect OC OS by modulating immune cells involved in immune escape (Vafaee et al., 2017; Shang et al., 2019; Colvin et al., 2020). Thus, we speculated that they influence OC by regulating B-cells, CD4^+^ T-cells, CD8^+^ T-cells, dendritic cells, macrophages, and neutrophils. Besides, highly expressed CD57^+^ NK cells and CD8^+^ T-cells are beneficial for OC prognosis (Henriksen et al., 2020). OC patients with high M1-polarized macrophage levels have better survival times (Macciò et al., 2020). These immune cells are involved in the occurrence and prognosis of OC (Oberg et al., 2019; Casanova-Acebes et al., 2020). Here, lncRNAs in the ceRNA network were significantly associated with multiple immune cells in OC, implying that lncRNAs in the ceRNA network participate in immune modulation of OC, thereby affecting its prognosis via multiple immune cells.

The main limitation of this study is the lack of more experimental validation of the functions of these lncRNAs in OC. Thus, our findings warrant validation using molecular approaches and large-scale clinical studies.

## Conclusion

We constructed a ceRNA network related to OC immunity and identified 4 lncRNAs (LINC00525, AL360004.1, TLR8-AS1, and LINC00402) as potential independent OC prognostic biomarkers. Furthermore, we show that these lncRNAs modulate multiple immune cells in OC.

## Data Availability Statement

Publicly available datasets were analyzed in this study. This data can be found here: https://portal.gdc.cancer.gov/, https:// gtexportal.org/, https://www.ncbi.nlm.nih.gov/geo/query/acc.cgi?acc=GSE26193, and https://www.ncbi.nlm.nih.gov/geo/query/acc.cgi?acc=GSE63885.

## Author Contributions

HL, XY, and HW designed the study. HL got the data, wrote, and reviewed the manuscript. HL and YB processed and analyzed the data. XY and HW supervised the critical revision of the manuscript. All authors have read and approved the final version of this manuscript.

## Conflict of Interest

The authors declare that the research was conducted in the absence of any commercial or financial relationships that could be construed as a potential conflict of interest.

## Footnotes

1 https://portal.gdc.cancer.gov/

2 https://gtexportal.org/

3 https://www.ncbi.nlm.nih.gov/gds/

4 https://cibersortx.stanford.edu/

5 http://www.mircode.org/

6 http://www.targetscan.org/

7 http://mirdb.org/

8 http://mirtarbase.mbc.nctu.edu.tw/

9 https://cytoscape.org/

10 http://ci.smu.edu.cn/genclip3/analysis.php

11 http://bio-bigdata.hrbmu.edu.cn/ImmLnc/
